# Analysis of risk factors of PICC-related bloodstream infection in newborns: implications for nursing care

**DOI:** 10.1186/s40001-021-00546-2

**Published:** 2021-07-23

**Authors:** Yan Hu, Yun Ling, Yingying Ye, Lu Zhang, Xiaojing Xia, Qianwen Jiang, Fang Sun

**Affiliations:** Department of Nursing, Children’s Hospital, School of Medicine, Zhejiang University, No. 3333 Binsheng Road, Binjiang District, Hangzhou City, Zhejiang Province China

**Keywords:** Newborns, PICC, CRBSI, Nurse, Care, Management

## Abstract

**Background:**

It is necessary to analyze the characteristics and risk factors of catheter-related bloodstream infection (CRBSI) in newborns with peripherally inserted central catheter (PICC).

**Methods:**

Newborns undergoing PICC catheterization in the neonatal department of our hospital from January 1, 2020 to January 31, 2021 were included. The characteristics of newborns with and without CRBSI newborns were compared and analyzed. Logistic regression analyses were performed to evaluate the risk factors of CRBSI in newborns with PICC.

**Results:**

Three hundred eighty-six newborns with PICC were included, of whom 41 newborns had the CRBSI, the incidence of CRBSI in newborns with PICC was 10.62%. There were significant differences regarding the birth weight, durations of PICC stay, 5-min Apgar score, site of PICC insertion of PICC between CRBSI and no CRBSI group (all *P* < 0.05), and there were no significant differences regarding the gender, gestational age, cesarean section, mechanical ventilation and length of hospital stay between CRBSI and no CRBSI group (all *P* > 0.05). *Escherichia coli* (26.08%) and *Staphylococcus aureus* (23.92%) were the most common CRBSI pathogens in newborns with PICC. Logistic regression analysis indicated that birth weight ≤ 1500 g (OR 1.923, 95% CI 1.135–2.629), durations of PICC stay ≥ 21 days (OR 2.077, 95% CI 1.024–3.431), 5-min Apgar score ≤ 7 (OR 2.198, 95% CI 1.135–3.414) and femoral vein insertion of PICC (OR 3.044, 95% CI 1.989–4.306) were the independent risk factors of CRBSI in neonates with PICC (all *P* < 0.05).

**Conclusion:**

For newborns with low birth weight, longer durations of PICC stay and femoral vein PICC insertion, they may have higher risks of CRBSI, and medical staff should take targeted measures to reduce the development of CRBSI.

## Background

Peripherally inserted central catheter (PICC) refers to the use of a catheter to puncture a vein in the peripheral vein, and the catheter goes directly to the large vein near the heart, providing convenience for neonatal fluid infusion. PICC has become an important life channel for neonates especially premature neonates in clinical treatment [1, 2]. The PICC application has the characteristics of simple operation, softness and easy observation [3]. It can stay in the body for a long time without repeated puncture. It provides a better infusion channel for neonatal treatment and is convenient for the infusion of important vasoactive drugs, intravenous nutrient solutions, antibiotics [4]. Therefore, PICC is widely used in neonates, especially premature infants.

Peripherally inserted central catheter is an important way of long-term intravenous infusion or nutritional support for newborns, but it may also have many complications. It has been reported that it is easy to cause catheter blockage, infection, mechanical phlebitis and other adverse complications due to improper care, of which the most serious one is catheter-related bloodstream infection (CRBSI) [5]. Previous studies [6, 7] have reported that the incidence of CRBSI in NICU varied from 7.25 to 13.78%. CRBSI not only prolongs the hospitalization time of newborns, but also may increase the mortality of newborns [8]. In order for PICC to be better used in newborns, it is very important to properly use cluster nursing interventions to prevent the occurrence of CRBSI. Therefore, we aimed at retrospectively analyzing the characteristics of neonates with PICC, to identify the risk factors of CRBSI in neonates with PICC, thereby providing reliable evidences to the management of PICC in clinical nursing care.

## Methods

### Ethical approval

In this study, all methods were performed in accordance with the relevant guidelines and regulations. Our study had been checked and approved by the ethical committee of medical research of our hospital (Approval Number: NU190128c), and written informed consents had been obtained from the guardians or relatives of included neonates.

### Neonates

We selected newborns who underwent PICC catheterization in the neonatal department of our hospital from January 1, 2020 to January 31, 2021 as the research populations. The inclusion criteria for newborns were as following: (1) newborns younger than 28 days old; (2) PICC insertion was conducted in our department, and no signs of infection such as fever, positive blood culture were found before the catheterization; and (3) the newborn’s family members were well informed and signed informed consent. The exclusion criteria for this study were: (1) newborns who underwent PICC insertion in other hospital; (2) newborns who were extubated within 48 h of PICC insertion; and (3) newborns whose family members refused to participate in this study.

### Peripherally inserted central catheter insertion

In this study, PICC insertions were performed by three PICC specialist nurses who had received professional theory and practice training. We selected 1.9 F catheter produced by Medical Components, Inc. DBA-Med Company to perform the PICC. The basilic vein was selected as the first choice for PICC puncture site, followed by the median cubital vein, axillary vein, and femoral vein. PICC operators were all personnel who had obtained the PICC specialist qualification certificate with professional training and have been engaged in clinical work. The PICC was placed in a laminar flow condition, and all the PICC insertion was conducted according to related standard procedures. After PICC catheterization, X-rays were used to confirm whether the catheter was in place.

### Catheter-related bloodstream infection diagnosis

The HP/PYP blood culture bottle was used to culture newborn blood samples, BACTEC 9240 blood culture instrument (USA) was used to test newborn blood samples, and the tip of the PICC catheter was placed in the blood agar culture medium (Becton, Dickinson and Company) for cultivation. Strain identification was analyzed with VITEK microbial automatic analyzer RC200 (France BioMérieux). In this study, the CRBSI was defined as the same colony cultured in peripheral blood and the tip of the PICC catheter after 48 h of catheter placement or within 48 h of extubation [9].

### Data collection

Two authors independently collected following information with unified forms, including gender, gestational age, birth weight, cesarean section, durations of PICC stay, 5-min Apgar score, mechanical ventilation, site of PICC insertion and the length of hospital stay.

### Statistical analysis

We used SPSS 23.00 software to perform statistical analysis. Measurement data were expressed as mean ± standard deviation, and comparison between groups was performed by *t* test. The count data were expressed as the number of cases or percentages, and the Chi-square test was used for comparison between groups. The factors with statistical significance in univariate analysis were further included in multivariate analysis, and related risk factors were analyzed by logistic regression model. In this study, *P* ≤ 0.05 was considered as the difference between the groups was statistically significant.

## Results

### The characteristics of included newborns

A total of 386 newborns with PICC were included, of whom 41 newborns had the CRBSI; the incidence of CRBSI in newborns with PICC was 10.62%. As indicated in Table 1, there were significant differences regarding the birth weight, durations of PICC stay, 5-min Apgar score, site of PICC insertion of PICC between CRBSI and no CRBSI group (all *P* < 0.05), and there were no significant differences regarding the gender, gestational age, cesarean section, mechanical ventilation and length of hospital stay between CRBSI and no CRBSI group (all *P* > 0.05).

**Table 1:** The characteristics of included newborns

Variables	CRBSI group (*n* = 41)	No CRBSI group (n = 345)	*t*/*χ*^2^	*P*
Gender (male/female)	24/17	211/134	1.215	0.107
Gestational age (weeks)	32.10 ± 4.58	33.04 ± 2.16	3.108	0.075
Birth weight (g)	1468.82 ± 212.14	1596.24 ± 243.92	42.137	0.012
Cesarean section	32 (78.05%)	260 (75.36%)	1.196	0.088
Durations of PICC stay (days)	24.18 ± 3.01	17.29 ± 3.25	3.122	0.031
5-min Apgar score	6.14 ± 2.06	7.83 ± 2.19	1.287	0.024
Mechanical ventilation	35 (85.37%)	288 (83.48%)	1.441	0.092
Site of PICC insertion				
Basilic vein	24 (58.54%)	257 (74.49%)	1.326	0.014
Cephalic vein	5 (12.19%)	32 (9.27%)		
Median elbow vein	4 (9.76%)	24 (6.96%)		
Axillary vein	3 (7.32%)	17 (4.93%)		
Femoral vein	5 (12.19%)	15 (4.35%)		
Length of hospital stay (days)	36.14 ± 7.21	35.58 ± 6.28	3.206	0.065

### Catheter-related bloodstream infection pathogens

As indicated in Table 2, of the 41 cases of CRBSI, 46 pathogens were detected in the bacteria culture. *Escherichia* *coli* (26.08%) and *Staphylococcus* *aureus* (23.92%) were the most common CRBSI pathogens in newborns with PICC.

**Table 2:** Distribution of CRBSI pathogens in neonates with PICC (*n* = 46)

Pathogens	Number	Proportion (%)
Gram-positive bacteria	19	41.31
*Staphylococcus* *aureus*	11	23.92
*Hemolytic* *streptococcus*	5	10.87
*Staphylococcus* *epidermidis*	3	6.52
Gram-negative bacteria	24	52.17
*Escherichia* *coli*	12	26.08
*Pseudomonas* *aeruginosa*	5	10.87
*Enterobacter* *cloacae*	3	6.52
*Klebsiella* *pneumoniae*	2	4.35
*Acinetobacter* *baumannii*	2	4.35
Fungus	3	6.52
*Candida* *albicans*	3	6.52

### The risk factors of CRBSI in neonates with PICC

The variable assignment of multivariate logistic regression is presented in Table 3. As indicated in Table 4, logistic regression analysis found that birth weight ≤ 1500 g (OR 1.923, 95% CI 1.135–2.629), durations of PICC stay ≥ 21 days (OR 2.077, 95% CI 1.024–3.431), 5-min Apgar score ≤ 7 (OR 2.198, 95% CI 1.135–3.414) and femoral vein insertion of PICC (OR 3.044, 95% CI 1.989–4.306) were the independent risk factors of CRBSI in neonates with PICC (all *P* < 0.05).

**Table 3:** The variable assignment of multivariate logistic regression

Factors	Variables	Assignment
CRBSI	*Y*	Yes = 1, no = 2
Birth weight (g)	*X*_1_	≤ 1500 = 1, > 1500 = 2
Durations of PICC stay (days)	*X*_2_	≥ 21 = 1, < 21 = 2
5-min Apgar score	*X*_3_	≤ 7 = 1, > 7 = 2
Femoral vein insertion of PICC	*X*_4_	yes = 1, no = 2

**Table 4:** Logistic regression analysis on the risk factors of CRBSI in neonates with PICC

Variables	*β*	S x̅	OR	95% CI	*P*
Birth weight ≤ 1500 g	0.204	0.211	1.923	1.135–2.629	0.022
Durations of PICC stay ≥ 21 days	0.818	0.126	2.077	1.024–3.431	0.036
5-min Apgar score ≤ 7	0.421	0.117	2.198	1.135–3.414	0.017
Femoral vein insertion of PICC	0.119	0.203	3.044	1.989–4.306	0.009

## Discussion

One of the most serious complications of PICC is CRBSI, which is associated with a longer length of hospital stay, increased medical costs, and elevated risk of death [10]. Although the current clinical care has been improved in the selection of PICC catheter materials, the qualification requirements of puncture personnel, puncture operation procedures and schedule maintenance specifications, CRBSI are still inevitable. Previous studies [11, 12] have reported the incidence of PICC-related CRBSI is between 8.11 and 12.35%. The results of this study have shown that the incidence of CRBSI in our department is 10.62%, which is consistent with the results of related studies. Moreover, we have found that birth weight ≤ 1500 g, durations of PICC stay ≥ 21 days, 5-min Apgar score ≤  7 and femoral vein insertion of PICC are the independent risk factors of CRBSI in neonates with PICC (all *P* < 0.05). Clinically, nursing intervention measures should be taken as soon as possible in response to these risk factors to reduce the onset of CRBSI in neonates with PICC.

Neonates have low immunity and poor resistance to infection, and they are a high-risk group of nosocomial infections. PICC placement can increase the risk of nosocomial infections [13]. A multi-center case–control study [14] have showed that after PICC catheterization for more than 2 weeks, the risk of CRBSI continues to increase with the extension of PICC stay. The defense function of newborns with a birth weight of ≤ 1500 g is imperfect, and newborns with Apgar score ≤ 7 are in poor physical condition, which will increase the risk of CRBSI [15, 16]. In response to the above risk factors, nurses should take appropriate measures as much as possible to reduce the occurrence of CRBSI, such as daily assessment of extubation indications, appropriate PICC care.

Previous study [17] has analyzed the bacterial spectrum of CRBSI in 125 hospitals in 26 European countries and found that Gram-positive bacteria accounted for 70.7%, Gram-negative bacteria accounted for 22.2%, and fungi accounted for 7.2%. In this study, Gram-positive cocci accounted for 41.31%, Gram-negative bacilli accounted for 52.17%, and fungi accounted for 6.52%. This is different from previous related reports [18, 19]. The reason may be related to the strict control of antibiotics use in newborns in our department, and secondly to the hand hygiene of medical staff. The supervision of the department is related to the daily secondary quality control. In recent years, with the strict control of the use of antibacterial drugs in hospitals, the clinical use of antibacterial drugs has become more and more standardized and strict. Effective management of antibacterial drugs reduces the production of drug-resistant bacteria, which has caused changes in the bacterial spectrum of catheter CRBSI in recent years [20]. The preventive use of fluconazole has also greatly reduced the rate of fungal infections [21].

Peripherally inserted central catheter is a tool for intravenous therapy. The qualification declaration and certification process, training and assessment, and qualification access of operating nurses or physicians should comply with the industrial management of intravenous infusion, which is conducive to promoting the professional development of intravenous infusion therapy [22]. The patients who use PICC catheters are mostly children or tumor patients. The elasticity of the blood vessels of those patients is generally poor. At the same time, the anatomical position of the blood vessels of some patients has a certain variation [23]. Therefore, the operator is required to have very skilled technology and rich experience. It has been reported that the experiences of PICC operators may be associated with the CRBSI [24, 25]. Therefore, all PICC operations in the clinic must be performed by personnel with relevant operating qualifications [26, 27].

At present, many hospitals do not have clear regulations on the duration of PICC stay. Studies [28, 29] have reported that regular replacement of the catheter does not reduce the risk of CRBSI. Studies [30–32] have reported that in NICU, PICC catheter indwelling time is an independent risk factor for the occurrence of CRBSI. In the first 18 days after PICC catheterization, the incidence of CRBSI increases by 14% per day, and 19–35 days after catheterization, the incidence of CRBSI will no longer increase, and the incidence of CRBSI will increase by 33% every day in the 36–60 days after catheterization. However, studies [33, 34] have also reported that prolonging the indwelling time of PICC catheters does not increase the incidence of CRBSI, which may be related to adequate nutritional support, reduction of other invasive procedures, and increased skin maturity in children. At the same time, studies [35–37] have reported that strict aseptic techniques and careful observation and care are the key to preventing CRBSI. The insertion site of the PICC has a great influence on the risk of CRBSI. The basilic vein is generally straight and thick. When the arms are perpendicular to the torso, basilic vein is the straightest and most direct way. The chances of mechanical phlebitis and ectopic catheterization are the lowest after puncture of the basilic vein, so the basilic vein should be the first choice [38, 39]. Several previous studies [40–42] have pointed that the femoral vein catheterization is prone to blood embolism, thus femoral vein should be avoided for PICC insertion. It must be noted that the data evaluation concerning femoral vein catheters are based on purely 5 incidences in this present study, which is far too low to make any reasonable inferences. Therefore, the data should be interpreted with caution and more studies on this issue are needed in the future.

This study has certain limitations. First, the sample size of this study is small; it may be underpowered to detect the potential risk factors. Secondly, there are many risk factors for CRBSI in newborns with PICC. This study is a retrospective study design and the analyzed factors included are limited. The experiences of surgeons or nurses may have significant influences on the PICC-related infection. In our study, all the three PICC specialist nurses had more than 5 years experience of PICC insertion, limited by the sample size, it may not have power enough to detect the group differences. Other factors such as aseptic operation during puncture and the application of antibiotics during hospitalization may also have an impact on the development of CRBSI [43, 44]. How to prevent the occurrence of CRBSI in newborns with PICC still needs further investigation.

## Conclusions

In conclusion, the incidence of CRBSI in newborns with PICC is rather high, for newborns with birth weight ≤ 1500 g, duration of PICC stay ≥ 21 days, 5-min Apgar score ≤ 7 and femoral vein insertion of PICC, they may have higher risks of CRBSI, early preventions and interventions targeted on those risk factors should be implemented. In-depth understanding of the risk factors of neonatal PICC-related CRBSI, mastering clustered nursing intervention measures for the management of PICC is an important guarantee to ensure the smooth completion of neonatal intravenous treatment and the reduction of CRBSI.

## Data Availability

All data generated or analyzed during this study are included in this published article.

## Abbreviations

CRBSI: Catheter-related bloodstream infection
PICC: Peripherally inserted central catheter
